# A Traction Control Strategy with an Efficiency Model in a Distributed Driving Electric Vehicle

**DOI:** 10.1155/2014/261085

**Published:** 2014-08-12

**Authors:** Cheng Lin, Xingqun Cheng

**Affiliations:** ^1^Beijing Co-Innovation Center for Electric Vehicles, Beijing Institute of Technology, Beijing 100081, China; ^2^National Engineering Laboratory for Electric Vehicles, Beijing Institute of Technology, Beijing 100081, China

## Abstract

Both active safety and fuel economy are important issues for vehicles. This paper focuses on a traction control strategy with an efficiency model in a distributed driving electric vehicle. In emergency situation, a sliding mode control algorithm was employed to achieve antislip control through keeping the wheels' slip ratios below 20%. For general longitudinal driving cases, an efficiency model aiming at improving the fuel economy was built through an offline optimization stream within the two-dimensional design space composed of the acceleration pedal signal and the vehicle speed. The sliding mode control strategy for the joint roads and the efficiency model for the typical drive cycles were simulated. Simulation results show that the proposed driving control approach has the potential to apply to different road surfaces. It keeps the wheels' slip ratios within the stable zone and improves the fuel economy on the premise of tracking the driver's intention.

## 1. Introduction

To address the two urgent issues nowadays of protecting the environment and achieving energy sustainability, it is of strategic importance on a global scale to replace oil-dependent vehicles with electric vehicles (EVs) [1–3]. Compared with internal combustion engine vehicles, electric vehicles with motors have many fascinating advantages, such as the quicker and more accurate torque generation, the easier measurement of motor torque [4], which provide a broad prospect for the vehicle dynamics control. Many types of drive system have been developed for electric vehicles by researchers in the last few years. Kim et al. studied the mode transition control for an internal combustion engine together with a motor driving type [5]. Liu et al. made a further research on driving control for electric vehicles with independently driven front and rear wheels [6]. Motors of the distributed driving electric vehicle are mounted directly in wheels or nearby wheels, which lead to a short and efficient transmission chain and compact structure, so it has been becoming an important research direction of electric vehicles [7].

The vehicle dynamics control of the distributed driving electric vehicle is one of the research focuses currently, which attracts lots of scholars' attention [8–10]. Ibrahim et al. provided a new load torque estimator to improve the stability of the traction drive system [11]. Gasbaoui et al. proposed a direct torque control strategy to ensure safety and stability, which are verified through all types of roads [12]. Athari et al. proposed a novel torque vectoring control strategy for an electric-drive vehicle with four in-wheel motors to assist a driver in handling a vehicle in unexpected conditions [13]. Chen et al. studied a fuzzy adhesion control method for four-wheel driven electric vehicle and the method was confirmed by the road experiment [14]. Kim et al. put forward a driving control algorithm for a 6WD/6WS vehicle equipped with 6 in-wheel motors to improve vehicle stability and maneuverability [15]. Several researchers also investigated the control strategies for improving vehicle energy economy [16, 17]. To improve the energy economy and the driving stability, the control strategy for the distributed driving electric vehicles is necessary and very important.

Focusing on a 4WD (4 wheels drive) distributed driving electric vehicle, this paper presented a driving control strategy to improve the driving safety and fuel economy. In order to ensure the driving safety during the emergency case, we used the sliding mode control strategy to guarantee the wheel slip rate within the stable zone. Apart from this case, an efficiency model is implemented to reduce the energy consumption. The efficiency model is built by an optimization method within a two-dimensional design space and the space contains the acceleration pedal signal and the vehicle speed. When building the efficiency model, to avoid the problem that online optimization methods have the limitations of heavy computation, we use the response surface techniques to develop a predictive model which can realize a real-time control through an offline optimization stream. The driving control strategy for different cases was verified through the simulation.

This paper is organized as follows. The vehicle dynamics model is built in Section 2, where the basic parameters of the vehicle are given. In Section 3, the driving control strategy with an efficiency model is designed. The simulation studies and analysis experiment are reported in Section 4 before conclusions are drawn in Section 5.

## 2. Vehicle Dynamics Model

### 2.1. System Configuration

**Figure 1:**
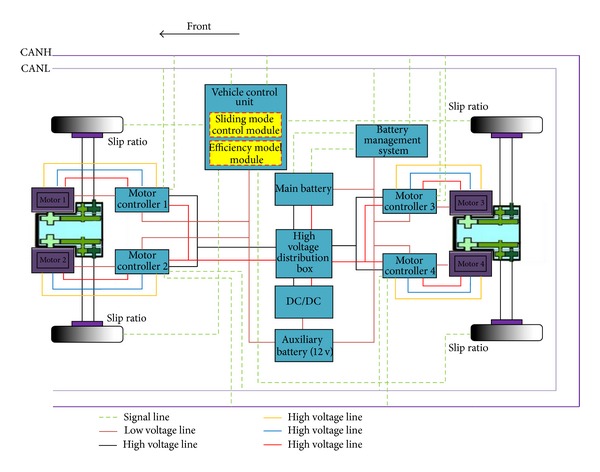
The vehicle structure.

The power system configuration of a distributed driving electric vehicle is shown in Figure 1.

It is a 4WD electric vehicle with four motors. Through high-voltage distribution box, the main battery, which was monitored by a battery management system directly, provides electric energy to each motor controller. The motor controller will invert the direct current to three-phase alternating current for motor according to the torque requirement command. In order to give the controllers and other low-voltage apparatus power supply, an auxiliary battery is equipped and it is charged by the main battery through DC/DC transformer. In addition, the vehicle communication is based on CAN (controller area net) bus. In longitudinal driving, the vehicle control unit will analyze and calculate each motor's torque demand of the electric vehicle in real time according to the control strategy. Then, the drive torque requirement of each motor will be transmitted to the CAN bus. After accepting the torque requirement signal from the CAN bus, the motor controller will change them into the three-phase alternating current to make the corresponding motor generate the actual drive torque. In the end, the motors drive the vehicle and the motor controllers give feedback to the CAN bus. In order to acquire the vehicle speed, the electric vehicle is equipped with a GPS.

The distributed driving electric vehicle specification is shown in Table 1.

### 2.2. Vehicle Longitudinal Dynamic Model

#### 2.2.1. Single-Wheel Model

**Figure 2:**
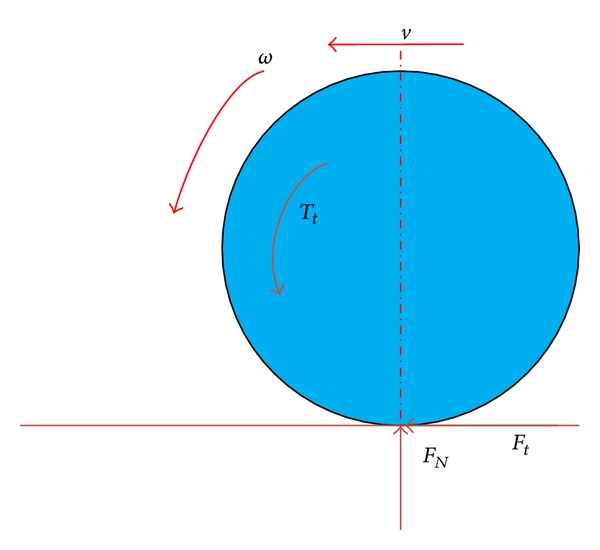
The single-wheel model.

In considering that each wheel can be controlled independently in the distributed driving electric vehicles, a single-wheel model is adopted. The model is shown in Figure 2. According to the vehicle dynamic, if the rolling resistance is ignored, the wheel motion equation can be defined as

(1) $$\begin{array}{c} I\dot{\omega }=T_{t}-F_{t}r, \end{array}$$

where *I* is the wheel rotational inertia, *F*_*t*_ means the road traction force of the wheel, *ω* denotes the wheel speed, *T*_*t*_ represents the traction torque of the wheel, and *r* is the radius of the wheel.

Furthermore, the slip ratio of the wheel can be described as

(2) $$\begin{array}{c} \lambda =\frac{\omega r-v}{\omega r}, \end{array}$$

where *λ* is the slip ratio of the wheel and *v* is the vehicle speed.

#### 2.2.2. Tire Model

The “Magic Formula” developed by Pacejka et al. is widely used as a modelling way in the dynamic simulation [18]. For the longitudinal dynamic motion, tire longitudinal force *F*_*t*_ can be simplified as

(3) $$\begin{array}{c} F_{t}=D\sin\left\{C\arctan\left[B\lambda -E\left(B\lambda -\arctan B\lambda \right)\right]\right\}, \end{array}$$

where *D*, *C*, *B*, and *E* denote the peak, shape, stiffness, and curvature factor, respectively. The above parameters can be obtained as follows:

(4) $$\begin{array}{c} C=1.65,\\ B=\frac{\left(a_{3}F_{N}+a_{4}\right)}{\left(\left(a_{1}F_{N}+a_{2}\right)C\times \exp\left(a_{5}F_{N}\right)\right)},\\ D=\left(a_{1}F_{N}+a_{2}\right)F_{N},\\ E=a_{6}F_{N}^{2}+a_{7}F_{N}+a_{8}, \end{array}$$

where *F*_*N*_ denotes the vertical load of the tire and *a*_*i*_  (*i* = 1,2,…, 8) is the fitting coefficient which can be obtained by Table 2.

#### 2.2.3. Driving Torque Model

**Figure 3:**
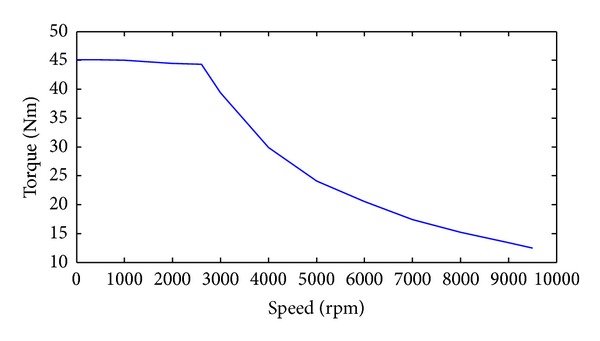
The peak torque versus motor speed curve.

Based on the peak torque of motors under a given motor speed, the required traction torque for the general driving case can be obtained as

(5) $$\begin{array}{c} T=4T_{\text{motor}\_\max}\times A_{\text{ps}}, \end{array}$$

where *T*_motor_max⁡_ is the peak torque of the motor under a given motor speed and it can be obtained by Figure 3; *A*_ps_ means the accelerator pedal signal.

#### 2.2.4. Motor Efficiency Model

**Figure 4:**
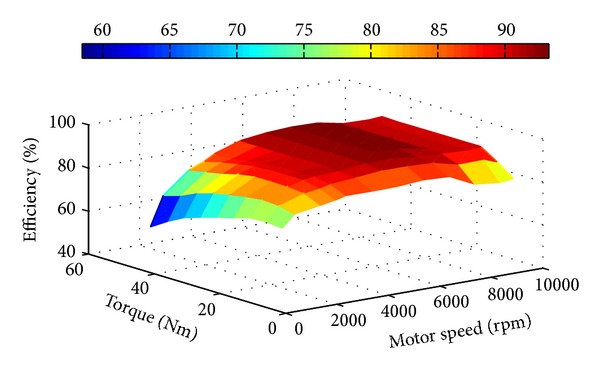
The efficiency map of the motor.

The motor efficiency *η*_motor_ comprising the motor controller efficiency shown in Figure 4 was measured by a dedicated dynamometer.

**Figure 5:**
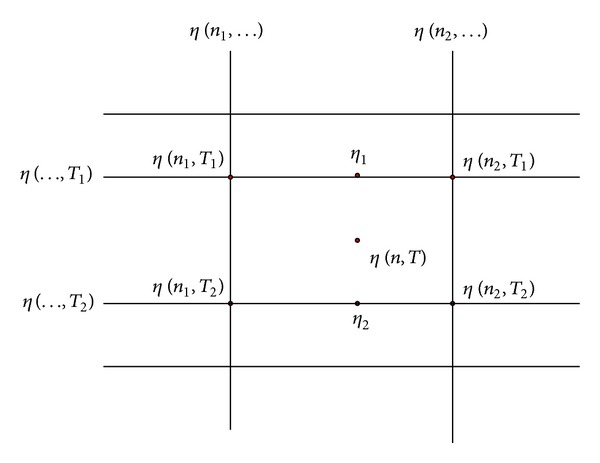
The binary Lagrange interpolation principle.

According to Figure 4, as long as the motor rotation speed *n* and the motor torque *T* have been calculated out, the corresponding efficiency values *η*_motor_ can be obtained from the test data table. The data which are not in the table can be calculated by using binary Lagrange interpolation method, as shown in Figure 5. The motor efficiency at any point can be calculated with the following formula:

(6) $$\begin{array}{c} \eta_{1}=\frac{n-n_{1}}{n_{2}-n_{1}}\eta \left(n_{2},T_{1}\right)+\frac{n_{2}-n}{n_{2}-n_{1}}\eta \left(n_{1},T_{1}\right),\\ \eta_{2}=\frac{n-n_{1}}{n_{2}-n_{1}}\eta \left(n_{2},T_{2}\right)+\frac{n_{2}-n}{n_{2}-n_{1}}\eta \left(n_{1},T_{2}\right),\\ \eta \left(n,T\right)=\frac{T-T_{1}}{T_{2}-T_{1}}\eta_{2}+\frac{T_{2}-T}{T_{2}-T_{1}}\eta_{1}. \end{array}$$

## 3. Driving Control Strategy and Algorithm

### 3.1. The Flow Chart of the Control Strategy

**Figure 6:**
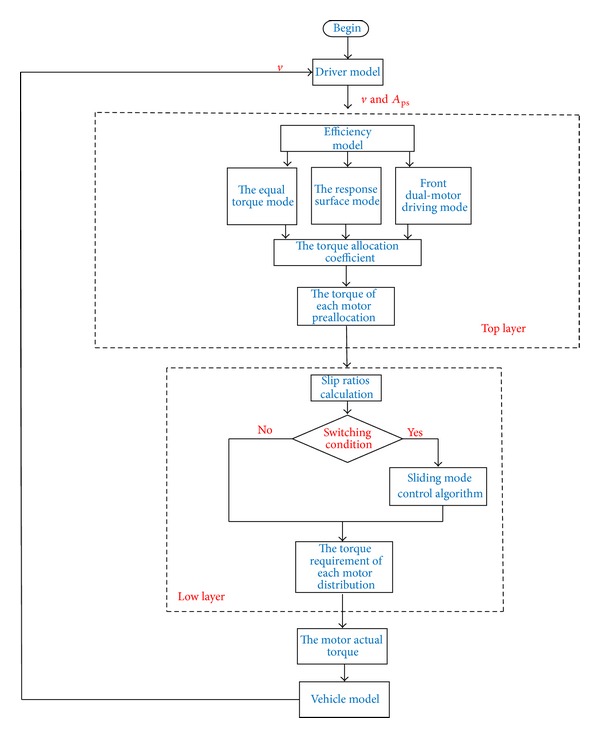
The traction control strategy.

This paper mainly investigated the control strategies on longitudinal driving scenario, which has two layers.  Figure 6 shows the hierarchical control strategy. The top layer is designed to obtain the reasonable motor torque's preallocation and the lower layer aims at guaranteeing the longitudinal stability and the required torque demand of the driver. According to the vehicle speed and the acceleration pedal signal, the top layer can obtain a motor torque preallocation scheme based on the efficiency model. Then the distributed driving torque will be delivered to the low layer. The low layer will calculate the slip ratio based on the torque preallocation value first and then judge whether it needs to adopt the sliding mode control algorithm. In the end it will give a torque requirement command to each motor obeying the principle of the equal torque between two wheels of the same axis.

### 3.2. The Top Layer

#### 3.2.1. The Efficiency Model

When the vehicle drives in the general case, the drive torque will be distributed by an efficiency model in this paper.

To maximize the driving efficiencies, the optimization method is usually applied to develop an efficiency model. However, it may involve the real-time problem during vehicle driving. So a response surface model technology was used in the efficiency model to realize the real-time control. The efficiency model was built in the two-dimensional design spaces that consist of the acceleration pedal signal and the vehicle speed based on offline optimization data.

(1)* The Optimization Mathematic Model*. To carry out the offline optimization stream, an optimization mathematic model was established to get the maximum of total driving efficiency.

The distributed driving electric vehicle (see Figure 1), which is the focus of this paper, has four motors. The overall output torque of the motors *T* is given by the following:

(7) $$\begin{array}{c} T=T_{1}+T_{2}+T_{3}+T_{4}, \end{array}$$

where *T*_1_, *T*_2_, *T*_3_, and *T*_4_ are the torques of motor 1, motor 2, motor 3, and motor 4, respectively.

Normally the two motor torques of each axis should be equal under the longitudinal dynamics model:

(8) $$\begin{array}{c} T_{1}=T_{2},\;\;T_{3}=T_{4}. \end{array}$$

As a consequence of the torque balance in (7) and (8), the operation points of the powertrain are fully determined by choosing the division of the motor torque between the front axis and the rear axis. So the traction force's allocation coefficient is defined as the torque split factor:

(9) $$\begin{array}{c} \alpha =\frac{T_{1}+T_{2}}{T}. \end{array}$$

When *α* equals 1, it means that the vehicle is in the front axis drive manner alone. Considering that the front axis driving manner alone is similar to the rear in its result, the allocation coefficient *α* can be limited from 0.5 to 1.

The overall efficiency of the powertrain system is obtained by the efficiency model of each motor from the experiment as follows:

(10) $$\begin{array}{c} \eta_{\text{total}}=\frac{\sum_{i=1}^{4}T_{i}n_{i}}{\sum_{i=1}^{4}\left(T_{i}n_{i}/\eta_{\text{motor},i}\right)}, \end{array}$$

where *T*_*i*_ and *n*_*i*_  (*i* = 1,2, 3,4) denote each motor speed and torque, respectively. *η*_motor,*i*_ denotes the efficient of each motor under the corresponding motor speed. *η*_total_ denotes the total efficient of the power motors system.

**Figure 7:**
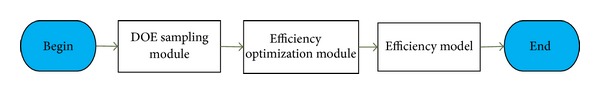
The offline efficiency optimization stream.

In considering that constraint, the optimization mathematic model can be expressed as follows:

(11) $$\begin{array}{c} \underset{\alpha \left(A_{\text{ps}},v\right)}{\max}\eta =\eta_{\text{total}}\left(A_{\text{ps}},v,\alpha \right),\\ T_{i}\leq T_{i,\max}\;\left(i=1,2,3,4\right),\\ 0\leq A_{\text{ps}}\leq 1,\\ 0.5\leq \alpha \leq 1,\\ 0\leq v\leq 120, \end{array}$$

where *T*_*i*,max⁡_  (*i* = 1,2, 3,4) denote the maximum output torques of every motor under a given motor speed, respectively.

(2)* The Offline Optimization Stream*. In accordance with the optimization mathematic model, an offline optimization was designed as shown in Figure 7. It can be mainly divided into three steps.

**Step 1 .**

Taking the acceleration pedal signal *A*_ps_ and the speed *v* as the continuous design space, a discrete sampling point set can be obtained in the DOE sampling module.

**Step 2 .**

Taking the sampling points as the input parameters, *α* as the optimization design variable, and the maximum driving efficiency *η*_total_ as the optimization target, the optimum value of *α* under each sampling point will be obtained by the efficiency optimization module.

**Step 3 .**

Based on the optimization data, which is obtained in the efficiency optimization module, create an efficiency model in which the response surface method is used. With regard to the efficiency model, *A*_ps_ and *v* are viewed as inputs, and the optimum value of *α* is viewed as output.

#### 3.2.2. DOE Sampling for the Continuous Input Design Space

**Figure 8:**
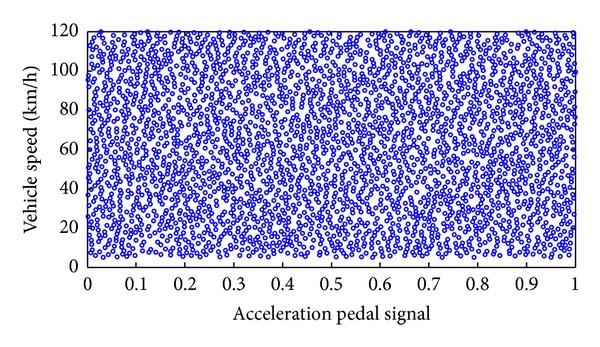
The sampling points in the design space.

As we can see from Figure 4, when the speed and torque of the motor are given, we can calculate the efficiency of this point by the efficiency map of the motor. Hence we take the vehicle speed and the acceleration pedal signal as the input design space, which can be deduced by the speed and torque of the motor. Considering the vehicle speed limit of the expressway, the maximum vehicle speed of the efficiency model is limited to 120 km/h and the acceleration pedal signal ranges from 0 to 1. Then the continuous input design space can be discrete as shown in Figure 8.

To improve the predictive precision of the efficiency model and evaluate the expected allocation coefficient, in this paper 3000 points were sampled in the design space (see Figure 8) using the optimization Latin hypercube design (Opt LHD) method. The Opt LHD can make the sampling point distribution more uniform and has a better space-filling performance than other experimental design methods [19].

#### 3.2.3. The Optimization and Analysis of the Traction Force Allocation Coefficient

From (9) we can see that the distributed coefficient has a linear relation with each motor torque. And as shown from Figure 4, the efficiency calculation is mainly a single peak problem. Therefore the Hooke-Jeeves technique is adopted during the optimization computation process, which is well suitable for both linear design spaces and nonlinear design spaces. Furthermore the approach is a direct search method and has a high rate of convergence [20].

**Figure 9:**
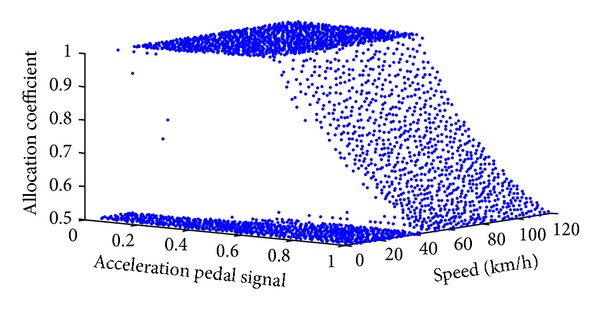
Allocation coefficients optimization results.

**Figure 10:**
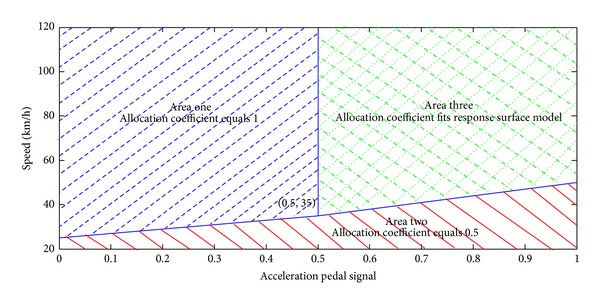
Design space area division.

Given a point in the design space, an optimal traction force distributed coefficient can be obtained by maximizing overall efficiency of the powertrain system shown in (11).  Figure 9 shows the optimization results of the allocation coefficient. To furthermore analyze the optimization results, we divide the design space into three areas using three colors according to the overall characteristics in Figure 9, as shown in Figure 10.

**Figure 11:**
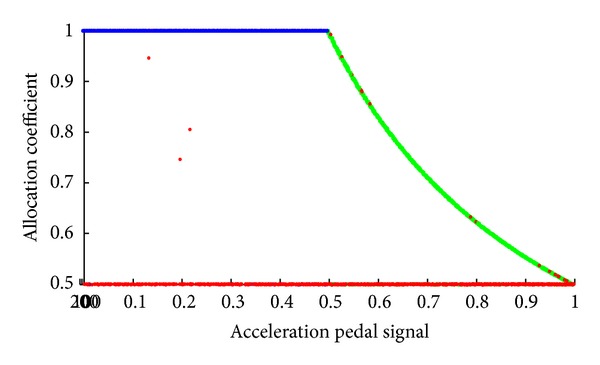
Optimization results side view.

**Figure 12:**
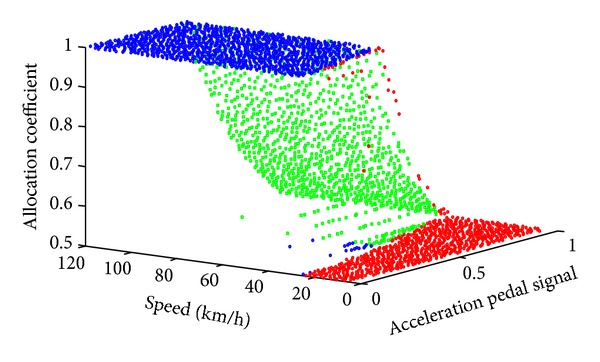
Optimization results right front view.

According to the design space area division results, we redraw the optimization results of the allocation coefficient using three colors, as shown in Figures 11 and 12. From the results above, we can set up an efficiency model with three zones. The first area is the blue one where the allocation coefficient equals 1; that is, only the motors of the front axis work. The second area is the red one where the allocation coefficient is 0.5 that means that each motor outputs equal torque. The third area is the green one where we should present a response surface model to predict the allocation coefficient based on the acceleration pedal signal and the vehicle speed.

#### 3.2.4. The Efficiency Model Design

As discussed above, a predictive model was proposed by using the RSM (response surface model) approach. RSM approximation is based on a polynomial fit via the least squares regression of the output parameters to the input parameters. Here the input parameters are the acceleration pedal signal and the vehicle speed and the output parameter is the allocation coefficient. The RSM obtained from the optimization data can be expressed as follows:

(12) α=−9.88783Aps+1.369829×10−6v+1.36306×101Aps2−1.28573×10−8v2−7.96798×10−7Apsv−9.27380Aps3+7.64492×10−11v3+2.49224Aps4−1.59573×10−13v4+3.53917.

### 3.3. The Lower Layer

The main target of the lower layer is to ensure the vehicle longitudinal stability and obtain the reasonable torque requirement of each motor distribution.

#### 3.3.1. The Switch Condition

The lower layer needs to judge the driving condition to realize the strategy switch. As we all know, according to the adhesion ratio versus slip ratio curves of the tires, if the slip ratio is lower than 15–20%, the vehicle will be at the general driving case. On the contrary, the vehicle will be at antislip control scenario. So, the switching condition is defined as the slip ratio exceeding 15%; that is, if the slip ratio is lower than 15%, it is at general driving case and efficiency model works; otherwise the sliding mode control algorithm will be activated to ensure the driving safety.

#### 3.3.2. The Sliding Mode Control Algorithm

When the vehicle driving in the emergency case, that is, the slip ratio being not lower than 15–20%, if the output traction torque still follows the required traction torque, the wheel will spin excessively. To ensure the driving safety, the traction torque should be limited and the sliding mode control strategy is used under such circumstance.

The sliding mode control strategy strives to control the slip ratio of the wheel to 16%, by which the maximum adhesion performance will be obtained. The switching function can be expressed as follows:

(13) $$\begin{array}{c} s=\lambda -\lambda_{0}+c\overset{t}{\underset{0}{\int }}\left(\lambda -\lambda_{0}\right)dt, \end{array}$$

where *λ*_0_ is the target value of the slip ratio, which is equal to 16%; *c* is theweighting parameter which denotes the slope value of the sliding curve.

The reaching law is defined as

(14) $$\begin{array}{c} \dot{s}=-\varepsilon \mathrm{sgn}\left(s\right), \end{array}$$

where *ε* denotes the reaching speed.

Taking a derivative of (13), we can give

(15) $$\begin{array}{c} \dot{s}=\dot{\lambda }+c\left(\lambda -\lambda_{0}\right). \end{array}$$

Taking a derivative of (2) gives

(16) $$\begin{array}{c} \dot{\lambda }=\frac{\left(v\dot{\omega }-\dot{v}\omega \right)}{\omega^{2}r}. \end{array}$$

Substituting (16), (1), and (14) to (15) gives

(17) $$\begin{array}{c} T_{t}=-\left(\varepsilon \mathrm{sgn}\left(s\right)+c\left(\lambda -\lambda_{0}\right)\right)\frac{I\omega^{2}r}{v}+\omega \dot{v}\frac{I}{v}+F_{t}\left(\lambda ,\dot{v}\right)r. \end{array}$$

#### 3.3.3. The Torque Requirement of Each Motor Distribution

If the slip ratio value is higher than 15% based on the motor torques preallocated in the efficiency model, the switch condition is satisfied and the sliding mode control strategy will be activated. In this case the torque preallocation value will be regulated based on the sliding mode controller and the equal torque principle of the wheels on the same axis. Then the torque distribution command of each motor will be transmitted to the motors accordingly.

## 4. Simulation Result and Analysis

Simulation studies have been carried out to verify the proposed control strategy under the assumption that the vehicle drives along straight road without road gradient.

### 4.1. The Simulation Analysis for the Efficiency Model

**Figure 13:**
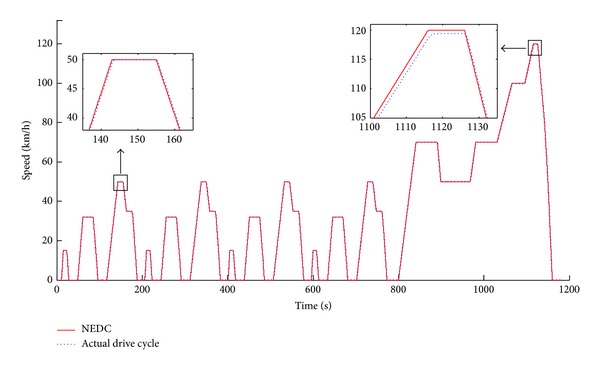
The NEDC and actual following DC.

**Figure 14:**
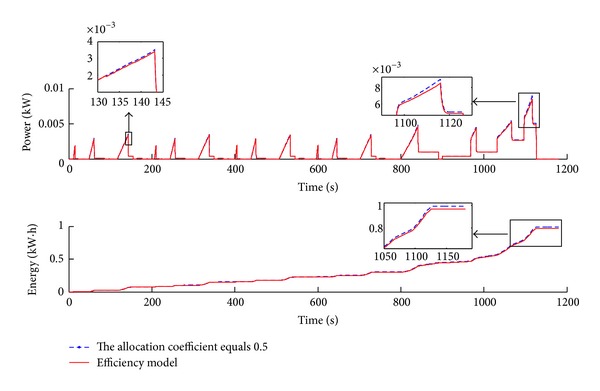
The time history of power and energy consumption over the NEDC.

**Figure 15:**
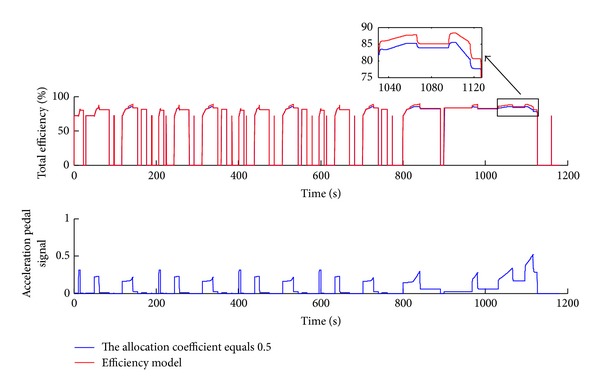
The time history of the total efficiency and acceleration pedal signal over the NEDC.

Figures 13, 14, and 15 show the simulation results of the efficiency model compared with even distribution strategy (allocation coefficient equals 0.5) for the general road scenario over the NEDC (New European Driving Cycle) condition.  Figure 13 illustrates that the actual following DC (Driving Cycle) in the simulation is well consistent with the NEDC, except that there is a little bit of tracking error in the high-speed section, but this does not affect the analysis of the simulation results.

Figure 14 illustrates the instant total power and the overall energy consumption improvement results. The simulation results in Figure 14 show that the proposed efficiency model can reduce the total power and overall energy consumption over the NEDC, especially in the high-speed zone.

Figure 15 shows the total efficiency contrast and the acceleration pedal signal over the NEDC. The simulation results indicate that the total efficiency has been improved under the efficiency model control strategy. The effect in the high-velocity zone of the driving cycle is obvious since the allocation coefficient is different from 0.5 mainly in those areas. Furthermore as shown in Figure 15, the acceleration pedal signal is mostly below 0.5, which conforms to the actual drive situation.

**Figure 16:**
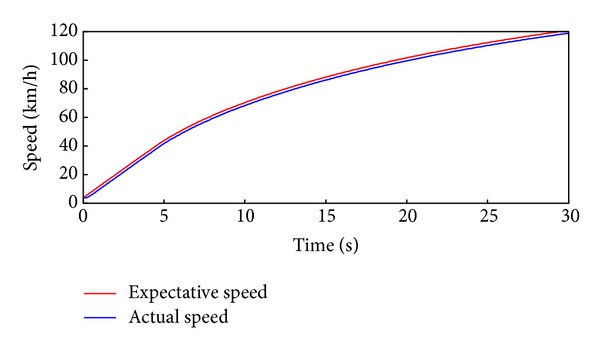
The acceleration driving cycle and actual following DC.

**Figure 17:**
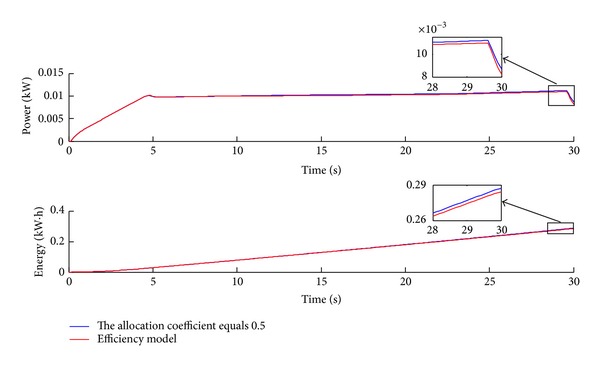
The time history of power and energy consumption over the acceleration DC.

**Figure 18:**
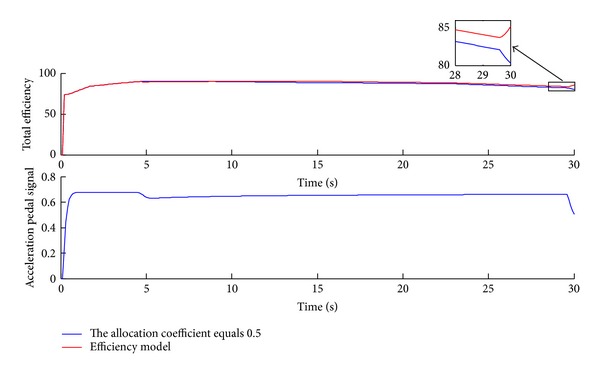
The time history of the total efficiency and *A*_ps_ over the acceleration DC.

To verify the response surface model, in which the acceleration pedal signal is larger than 0.5, the simulation of 30 seconds acceleration to 120 km/h has been conducted. As shown in Figures 16, 17, and 18, the simulation results are similar to the results over the NEDC. The instant power and energy consumption under the efficiency model control strategy are lower than under even distribution strategy, and the total efficiency is also higher. Furthermore, the acceleration pedal signal is between the interval of 0.5 and 0.7 in which the response surface model of the efficiency model works after 5 seconds or so.

Table 3 lists the simulation results of energy economy improvement. Compared with even distribution strategy, the proposed efficiency model control strategy can decrease energy consumption by 2.3% in the NEDC situation and by 1.1% in the 0~120 km/h acceleration case. The total average efficiency is 81.12% under the even distribution strategy and 81.89% under the efficiency model over the NEDC. And the total average efficiency under the efficiency model over the acceleration case is also higher than under the even distribution strategy as shown in Table 3. Therefore, the efficiency model control strategy can improve energy economy compared with the even distribution strategy.

### 4.2. The Simulation Analysis for the Sliding Mode Control Algorithm

#### 4.2.1. The Road Simulation Parameters

**Figure 19:**
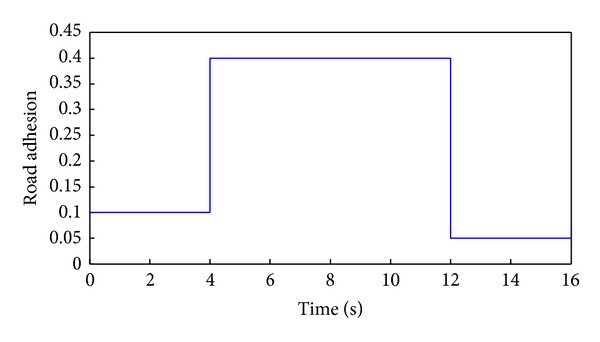
Adhesion coefficients of road surfaces.

The peak adhesion coefficient at each wheel represents the road adhesion conditions. This paper sets the surface parameters at each wheel of different road surfaces as shown in Figure 19. The adhesion coefficients of high adhesion road, low adhesion, and uniform surface are constant and the coefficients of joint road represent an adhesion step change surface.

#### 4.2.2. The Simulation Results and Analysis

**Figure 20:**
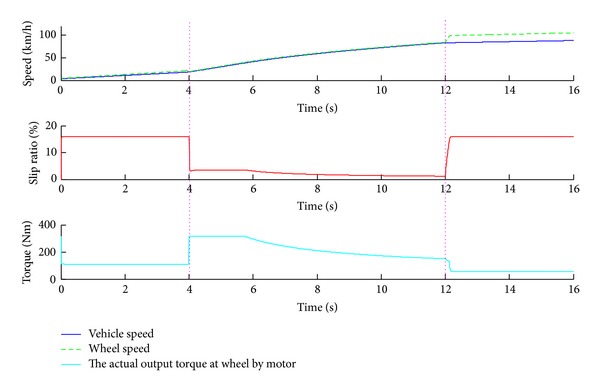
The simulation results of joint road surface.

Figure 20 shows the simulation results of sliding mode control strategy on the joint road surface which has three sections. As shown in Figure 19, the adhesion coefficients of the road sections are 0.1, 0.4 and 0.05 in sequence. First the distributed driving electric vehicle runs on the road whose adhesion is 0.1. When the slip ratio is higher than 15%, the sliding mode control strategy works and the slip ratio converges to 16%. While the vehicle comes from the first road section to the second road section whose adhesion is 0.4 at the fourth second, the slip ratio is lower than 15%. Then the sliding mode control strategy quits and the motor torque distribution complies with the efficiency mode control strategy. Since the vehicle reenters the low adhesion road surface at the twelfth second and the slip ratio is greater than 15%, the sliding mode control strategy resumes and the slip ratio also converges to 16% according to the setting value.

## 5. Conclusions

1. A traction control strategy, including an efficiency model control strategy for the general road surface scenario and the sliding mode control strategy for the emergency antislip control scenario, has been studied.
2. Several simulation experiments have been carried out to verify the efficiency model control strategy on the general road surface scenario. The simulation results demonstrated that the efficiency model control strategy could improve the vehicle fuel economy comparing with the even torque distribution strategy. It can decrease energy consumption by 2.3% in the NEDC situation and by 1.1% in the 0~120 km/h acceleration DC case under the proposed efficiency model control strategy.
3. Aiming at the emergency driving scenario, the sliding mode control strategy has been analyzed on the joint road surface. The simulation results indicate that the slip ratios were preferably controlled to 16% for different adhesion road surface change cases.

## Figures and Tables

**Table 1:** Basic parameters of the vehicle.

Parameters	Symbols	Units	Values
Gross mass	*m*	kg	1350
Wheelbase (front, rear)	(*l*_f_, *l*_r_)	m	(1.085, 1.386)
Track width (front, rear)	(*d*_f_, *d*_r_)	m	(1.429, 1.422)
Centroid height	*h*_g_	m	0.48
Windward area	*A*	m^2^	1.895
Air drag coefficient	*C*_D_	—	0.34
Wheel rolling radius	*r*	m	0.281
Rolling resistance coefficient	*f*_r_	—	0.018
Wheel rotational inertia	*I*	kg*·*m^2^	0.87
Reducer ratio	*i*_0_	—	7.013
Motor peak/rated power	*P*_max⁡_/*P*_N_	kW	12.5/7.5
Motor maximum/rated speed	*n*_max⁡_/*n*_N_	rpm	9500/4000
Motor peak/rated torque	*T*_max⁡_/*T*_N_	N*·*m	45/18

**Table 2:** The fitting coefficients of the “Magic Formula”^∗^.

*a*_1_	*a*_2_	*a*_3_	*a*_4_	*a*_5_	*a*_6_	*a*_7_	*a*_8_
−21.3	1144	49.6	226	0.069	−0.006	0.056	0.486

*a_*i*_ (*i* = 1,2,…, 8) is the fitting coefficient required to solve the longitudinal tire force, respectively, in the “Magic Formula” developed by Pacejka et al. [18].

**Table 3:** Energy consumption result.

Strategy	Even distribution strategy		The efficiency model		
Situation	Energy consumption	Total average efficiency	Energy consumption	Total average efficiency	Improvement
NEDC	0.9916 (kw*·*h)	81.12%	0.9689 (kw*·*h)	81.89	2.3%
Acceleration DC	0.2876 (kw*·*h)	87%	0.2845 (kw*·*h)	87.86%	1.1%
